# Functional marker development of miR1511-InDel and allelic diversity within the genus Glycine

**DOI:** 10.1186/s12864-015-1665-3

**Published:** 2015-06-18

**Authors:** Nang Myint Phyu Sin Htwe, Zhong-Qin Luo, Long-Guo Jin, Brian Nadon, Ke-Jing Wang, Li-Juan Qiu

**Affiliations:** The National Key Facility for Crop Gene Resources and Genetic Improvement (NFCRI), Institute of Crop Science, Chinese Academy of Agricultural Sciences, 100081 Beijing, China; Institute of Plant Breeding, Genetics, and Genomics, University of Georgia, Athens, GA USA

**Keywords:** Soybean, miRNA, Genetic diversity, Genetic marker

## Abstract

**Background:**

Single-stranded non-protein coding small RNAs, 18–25 nucleotides in length, are ubiquitous throughout plants genomes and are involved in post-transcriptional gene regulation. Several types of DNA markers have been reported for the detection of genetic diversity or sequence variation in soybean, one of the most important legume crops in worldwide for seed protein and oil content. Recently, with the available of public genomic databases, there has been a shift from the labor-intensive development of PCR-based markers to sequence-based genotyping and the development of functional markers within genes, often coupled with the use of RNA information. But thus far miRNA-based markers have been only developed in rice and tobacco. Here we report the first functional molecular miRNA marker, miR1511-InDel, in soybean for a specific single copy locus used to assess genetic variation in domesticated soybean (*Glycine max* [L.] Merr) and its wild progenitor (*Glycine soja* Sieb. & Zucc.).

**Results:**

We genotyped a total of 1,669 accessions of domesticated soybean (*G. max*) and its wild progenitor *G. soja* which are native throughout the China and parts of Korea, Japan and Russia. The results indicate that the miR1511 locus is distributed in cultivated soybean and has three alleles in annual wild soybean. Based on this result, we proposed that miR-InDel marker technology can be used to assess genetic variation. The inclusion of geo-reference data with miR1511-InDel marker data corroborated that accessions from the Yellow River basin (Huanghuai) exhibited high genetic diversity which provides more molecular evidence for gene diversity in annual wild soybean and domestication of soybean.

**Conclusions:**

These results provide evidence for the use of RNA marker, miRNA1511-InDel, as a soybean-specific functional maker for the study of genetic diversity, genotyping of germplasm and evolution studies. This is also the first report of functional marker developed from soybean miRNA located within the functional region of pre-miRNA1511.

**Electronic supplementary material:**

The online version of this article (doi:10.1186/s12864-015-1665-3) contains supplementary material, which is available to authorized users.

## Background

Soybean (*G. max*) is one of the most economically important global crops, providing not only proteins and vegetable oils for human beings but also feed for livestock and biofuel feedstock [1]. Moreover, it is one of the most commonly cultivated crops worldwide and is known for its capacity to fix atmospheric nitrogen through symbioses with soil-borne microorganisms [2] and has nutraceutical properties such as isoflavones, saponins and tocopherols [3]. Molecular markers are useful tools for basic research and plant breeding to determine the order of genes along the chromosome, detect genetic variability and diversity, construct genetic maps, and track individuals or lines carrying particular genes/loci [4]. In addition, they has been used for studies in molecular ecology, developmental biology, conservation biology, phylogenetics and systematic, as well as in diagnostic of forensic, paternity and diseases assessment [5].

The genetic linkage map in humans using restriction fragment length polymorphism (RFLP) was the one of the first molecular marker applications used in the detection of DNA polymorphism [6]. With the innovation of PCR technology, several alternative DNA marker techniques such as random amplified polymorphic DNA (RAPD) [7], amplified fragment length polymorphisms AFLP [8], simple sequence repeats (SSR) [9], and single-nucleotide polymorphisms (SNP) [10] were developed. In recent years, technological advances have contributed to advancements in every aspect of molecular marker techniques making them technically initiating a trend away from random DNA markers to functional markers due to rapid growth in genomic research [11]. In soybean, various types of DNA-based markers have been used for linkage mapping [12–14], analysis of genetic variability and diversity [15, 16], and identify lines carrying specific genes [17]. Recently, with the availability of genome sequencing in soybean, it has become relatively easy to find single-nucleotide polymorphisms (SNPs) and small insertions/deletions (InDels) which are attractive mapping tools as compared to many other markers types. For example, *de novo* assembly a pan-genome for wild soybean indicated that three InDels in annual wild soybean (*G. soja*) may be responsible for changing the growth habit from the twining in *G. soja* to the erect type in *G. max* [18]. Moreover, from the same data set, 0.50 to 0.77 million InDels were detected in *G. soja* as compared to *G. max* [18]. In addition, the usefulness of InDel markers are supported by their wide distribution across the genome and that they are easily assayed by PCR using flanking sequences [19].

MicroRNAs (miRNAs) are a new type of abundant small RNAs 18–25 nucleotides in length [20], and play key roles in plant development [21, 22], response to nutrient deficiency [23] and other abiotic stresses [24, 25]. MicroRNAs can be phylogenetically conserved and silence gene expression at the post-transcriptional level by either cleaving or inhibiting translation of a target mRNA via complementation between the miRNA and target mRNA [20, 26, 27]. Recent developments of next generation or deep sequencing technologies have resulted in an increase in microRNA discovery, miRNA detection and analysis [28]. The miRNA Registry Database as of release 21, June 2014 contained 28,645 stem loop miRNAs with 35,825 mature miRNA sequences from 223 species of which there were 573 stem loop miRNAs with 639 mature miRNA sequences for cultivated soybean and 13 stem loop miRNAs with 13 mature miRNA in wild soybean (http://www.mirbase.org/). With the increase in miRNA sequences, it has become possible to develop miRNA-based markers, which is a new type of marker with easy and efficient detection. Using multi-mapped small RNA sequences, inter small RNA polymorphism (iSNP) marker technology was developed for mapping and fingerprinting in species with extremely low genetic diversity [29]. In addition, small RNA-based SSR markers were developed for genetic diversity analysis between salt tolerant and sensitive rice genotypes [30].

miR1511 is a novel small RNA first identified in soybean roots by deep sequencing [31] followed by discovery during stress response in different organs and growth conditions in *Phaseolus vulgaris* [32], in root and nodules of M*edicago truncatula* [33], and in various tissue of *Vitis vinifera* [34]. Base on bioinformatics information, the precursor of pre- miR1511, a 129 bp sequence, is located in an intergenic region on chromosome 18, 10,405 bp downstream of *Glyma18g19410.1* and 12,789 bp upstream of *Glyma18g19420.1* in William 82. In our previous study, we confirmed the existence of miR1511 and identified its target gene [35]. In this study, we developed a miR1511-InDel marker, which was used to investigate genetic diversity in a large sample of *G. max* and *G. soja*. These results provide a reference for miRNA1511-InDel as a new functional marker type for the study of genetic diversity, gene mapping and molecular breeding.

## Results

### Functional marker for miR1511/miR1511* allelic variation

**Fig. 1:**
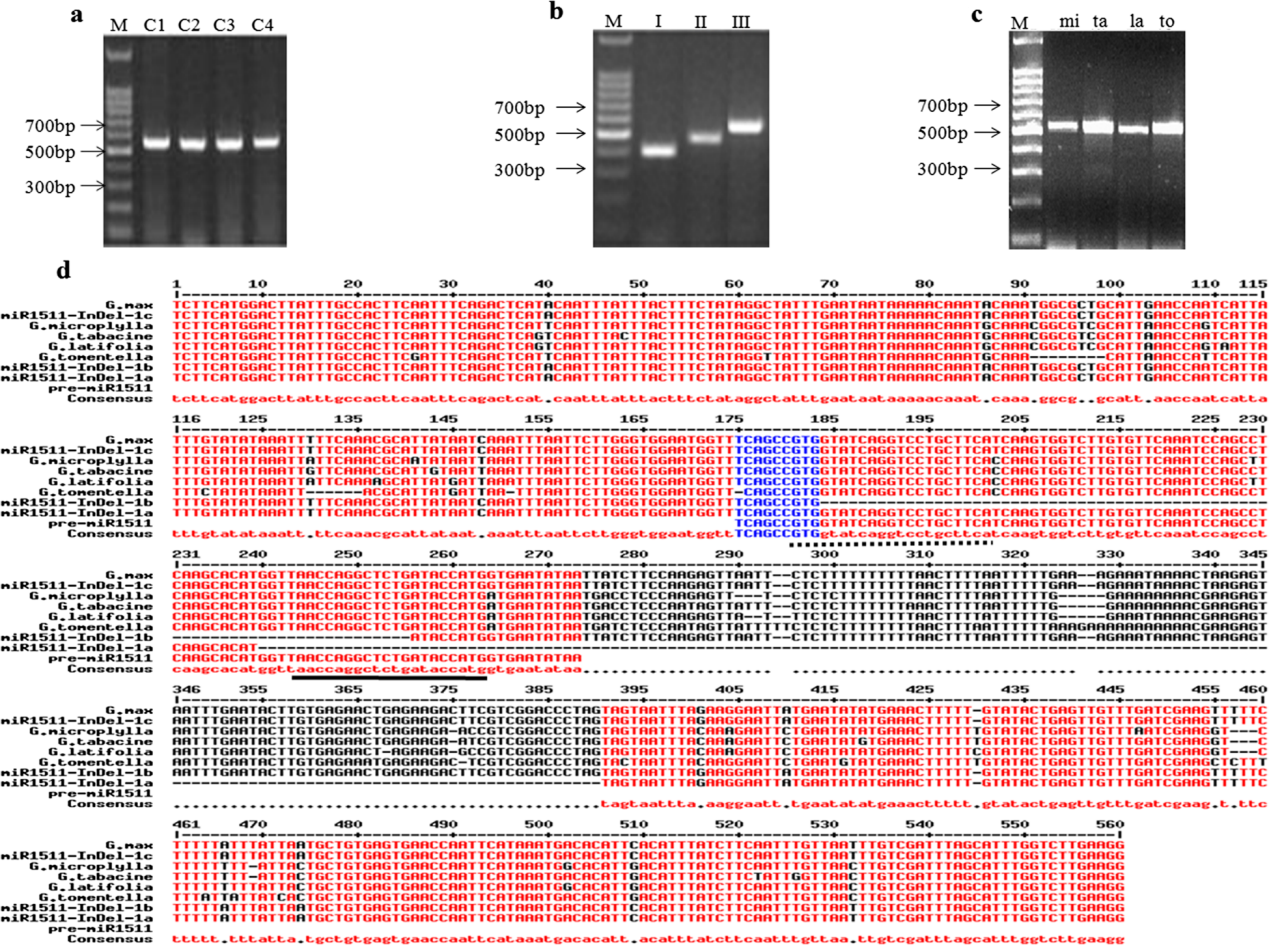
Amplification of MIR1511 locus by PCR and sequence validation of miR1511 within the genus *Glycine.* Amplification of MIR1511 locus by PCR and sequence validation of miR1511 within the genus *Glycine* (**a**) cultivated soybean, (**b**) annual wild soybean, (**c**) perennial wild soybean (**d**) sequence variation of miR1511 among four accession from subgenus Glycine and four accessions from subgenus Soja. C1-C4: different accession of cultivated species, I: *miR1511-InDel-1a*, II*: miR1511-InDel-1b*, III: *miR1511-InDel-1c*, mi: *G. microplylla*,ta:*G. tabacine*,la:*G. latifolia*, to: *G. tomentella*, full line: miR1511, dotted line: miR1511*

To determine allelic variation of soybean subgenus *G. glycine* and *G. soja*, we developed an Insertion/Deletion marker for miR1511/miR1511* (miR1511-InDel) in the flanking region of stem loop miR1511. We PCR amplified sequences that contained the pre-miR1511 locus and found three fragments sizes ranging from 400 to 600 bp. The result showed there were no differences in the size of amplified products in cultivated and perennial wild soybean (Fig. 1a, c), while there were three allelic variants in annual wild soybean (Fig. 1b). The amplified products from the cultivated, annual and perennial wild soybean accessions were verified by DNA sequencing. Sequencing results revealed that the soybean miR1511 mature sequence and miR1511* were found in *G. max* and perennial wild soybean (*G. microplylla, G. tabacine, G. latifolia,* and *G. tomentella)*. There were three types of allelic variants located within the stem loop of miRNA in annual wild soybean (named *miR1511-InDel-1a*, *miR1511-InDel-1b*, and *miR1511-InDel-1c*) (Table 1). *MiR1511-InDel-1a* had a 147 bp deletion including the complete mature sequence of miR1511. *MiR1511-InDel-1b* had a 71 bp deletion that included parts of both the mature miR1511 and the miR1511* sequence. *MiR1511-InDel-1c* included both mature miR1511 and miR1511* sequences and was identical to cultivated soybean (Fig. 1d). In order to know the feasibility and common of this type of miRNA-InDel marker, 573 stem loop miRNAs with 639 mature miRNA sequences were downloaded from miRBase (http://www.mirbase.org/) and multi aligned with seven *G. soja* accessions (A to G) in pan-genome [18]. There were more than 10 % (107/639 mature miRNA) of this type of miRNA-InDel markers. Interestingly, miR4387e and miR4399 have been found to have similar variation as miR1511-InDel that deletion in Yellow river region.

**Table 1:** Types of allelic variation in G. Soja

No.	Name of allele	Difference
1	*miR1511-Indel-1a*	Deletion of 147 bp including complete miR1511 mature sequence
2	*miR1511-Indel-1b*	Deletion of 71 bp including partial of miR1511* (18 bp deletion) and miR1511 (12 bp deletion) mature sequence
3	*miR1511-Indel-1c*	No InDel and have complete miR1511* (21 bp) and miR1511 (21 bp) mature sequence

### Phylogenetic analysis of miR1511

**Fig. 2:**
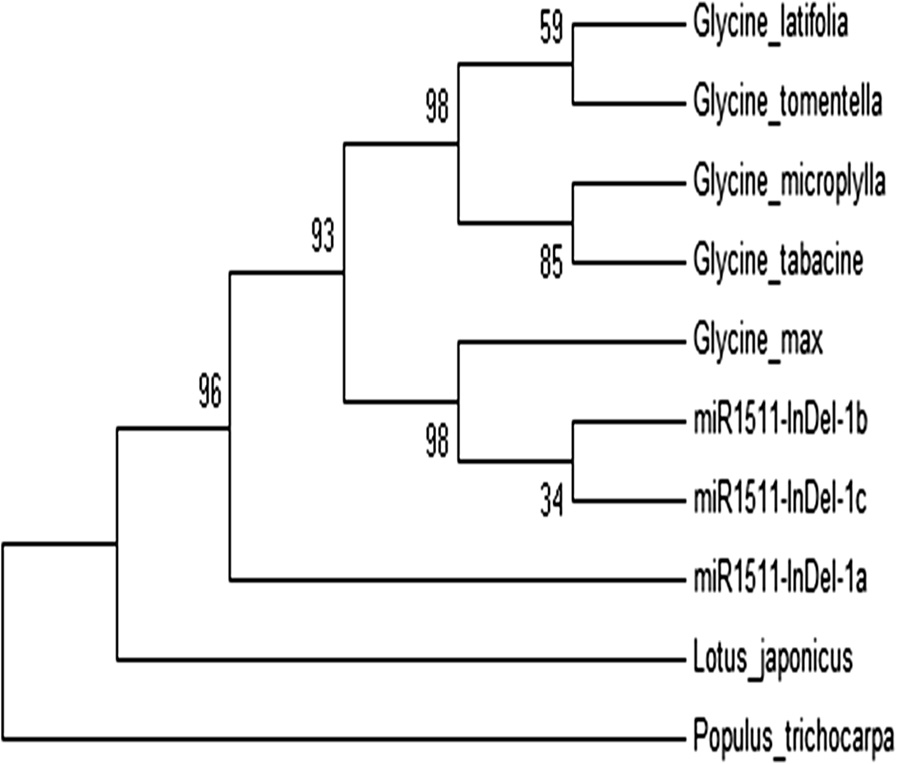
Phylogenetic analysis of miR1511. Tree constructed from a complete deletion alignment using MEGA 4.0 and the maximum likelihood method with 1,000 bootstrap replicates. The numbers at the nodes represent bootstrap support for the given relationships

In this study, we analyzed including an average 250 bp of 5' and 3' flanking sequences of pre- miR1511 in other crops using a low stringency BlastN search with the precursor sequence of soybean miR1511 as a query. The resulting output sequences were inspected for the presence of the mature sequence and then their secondary structures were predicted using the RNAfold program (http://mfold.rna.albany.edu/?q=mfold) (Additional file 1: Figure S1). Except for *G. max*, the mature sequence of miR1511 is not in the stem region in any legume species including *M. truncatula*, *L. japonica* and two out group species, *P. trichocarpa* and *V. vinifera* (Additional file 2: Figure S2). An un-rooted tree was constructed using the mature sequences of miR1511 among the subgenus Glycine, and two as outgroup which were not in stem region using the maximum likelihood (ML) method. The observed tree suggested that subgenus Glycine (*G. microplylla, G. tabacine, G. latifolia and G. tomentella*) and Soja, including cultivated soybean and annual wild soybean, group into one clade with a bootstrap value of 93 % with the exception of *miR1511-InDel-1a. G. soja* was divided into two groups based on presence or complete deletion of miR1511 sequence. *G. soja* with either *miR1511-InDel-1b* or *miR1511-InDel-1c* were grouped with *G. max* with a bootstrap value of 98 % and *G. soja* with *miR1511-InDel-1a* was outside the clade (Fig. 2).

### Allelic diversity of InDel marker miR1511

**Fig. 3:**
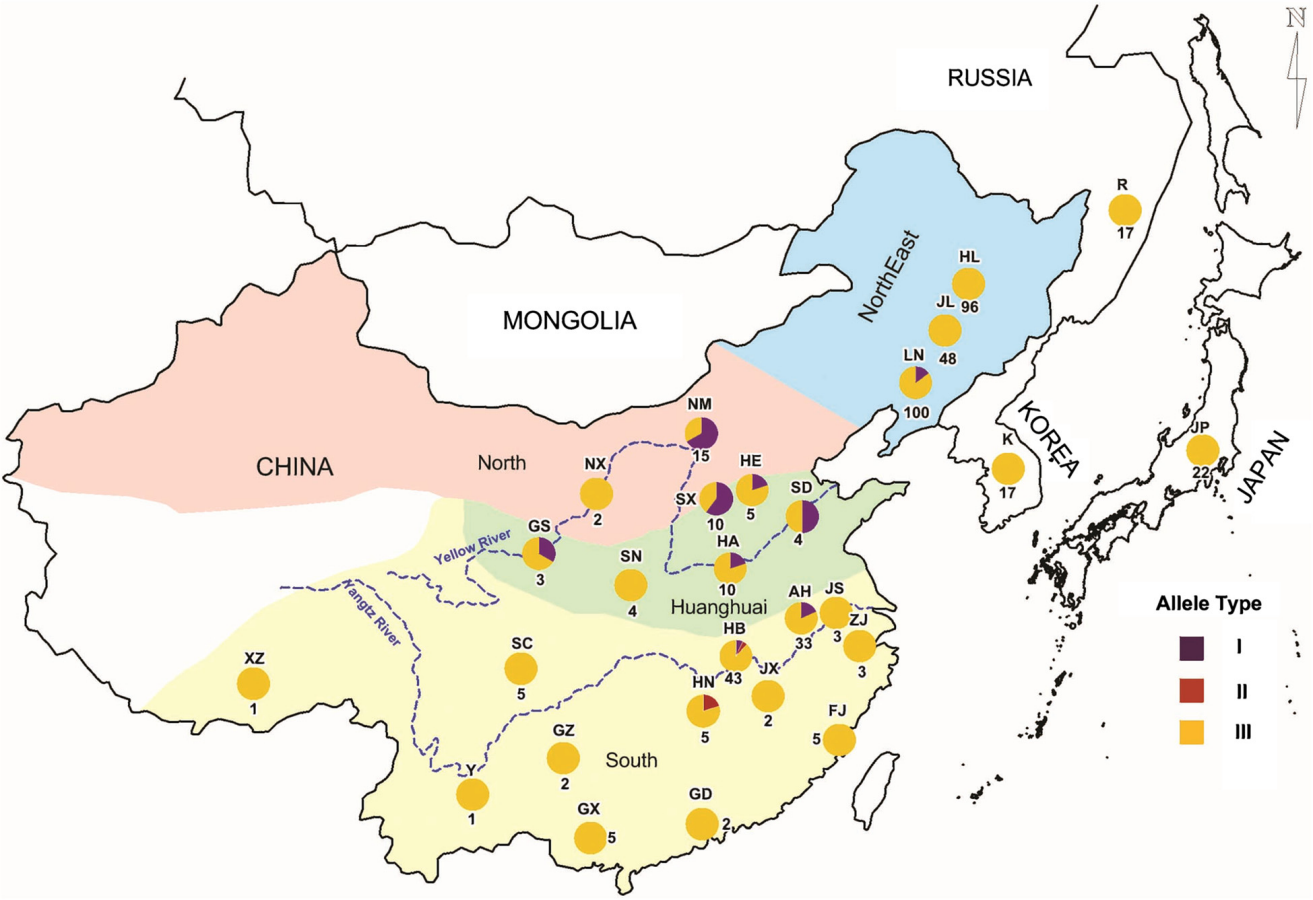
Geographic distributions of samples (*G.soja*) used in this study. Each circle represents a different province in China and the country out of China. The percentage of each allele of *G. soja* is designated by pie chart on the map. The total number of samples per province is indicated by the number. I: *miR1511-InDel-1a*, II: *miR1511-InDel-1b*, III: *miR1511-InDel-1c*, HL: Heilongjiang, JL: Jilin, LN: Liaoning, NM: Neimengu, NX: Ningxia, HE: Hebei, SX: Shanxi, SD: Shangdong, HA: Henan, SN: Shannxi, GS: Gansu, JS: Jiangsu, AH: Anhui, ZJ: Zhejiang, HB: Hubei, JX: Jiangxi, HN: Hunan, FJ: Fujian, GZ: Guizhou, SC: Sichuan, GX: Guangxi, Y: Yunnan, XZ: Xizang, GD: Guangdong

In order to validate the conserved mature miR1511 and miR1511* within *G. max*, and variation in *G. soja*, we used a total of 1,206 accessions of cultivated soybean (*G. max*) including a minicore collection representing four major soybean growing areas of China and 463 accessions of annual wild soybean (*G. soja*) from three regions of China, Japan, Korea and Russia. All cultivated soybean accessions contained mature miR1511 and miR1511* sequences (data not shown). As mentioned previously, there were three types of alleles in *G. soja. MiR1511-InDel-1c* was the most abundant and distributed throughout China, Japan, Korea and Russia, just as in the cultivated soybean accessions (Fig. 3). However, *miR1511-InDel-1a* containing a complete deletion of mature miR1511 was found mostly in accessions collected along the Yellow River basin including Gansu (GS), Hebei (HE), Henan (HA), Inner Mongolia (NM), Shandong (SD), and Shanxi (SX); whereas, *miR1511-InDel-1b,* with a partial deletion of both the mature miR1511 and miR1511* sequence, was found in Hunan (HN) and Hubei (HB) provinces (Fig. 3). Although many accessions were analyzed from the Northeast region of China, e.g. Heilongjiang (HL) and Jilin (JL) provinces, there was only one allele, *miR1511-InDel-1c*. Incidentally, accession *G. soja* D from Yellow River basin (Huanghuai), one of seven *G. soja* accession sequenced as part of a pan-genome, contained the *miR1511-InDel-1a* allele [18] (Additional file 3: Figure S3). The diversity index was highest in Yellow River basin (Huanghuai) followed by South region of China and Northeast region of China (Table 2). There was no genetic diversity for this locus in accessions of Korea, Japan and Russia. These results provide Yellow River basins (Huanghuai) is the original center for miR1511 locus diversity based on molecular evidence of allelic diversity of miR1511-Indel.

**Table 2:** Analyses of genetic variance based on miR1511-InDel marker on different classification/Region of soybean

Classification/Region	No. of Accessions	% of different type allele			Diversity index
		*miR1511-InDel-1a*	*miR1511-InDel-1b*	*miR1511-InDel-1c*	
Subgenus Glycine	4	0.00	0.00	100.00	0.00
Subgenus Soja					
*G.max*	1206	0.00	0.00	100.00	0.00
*G.soja*	463	9.94	0.65	89.42	0.36
Region					
China					
North east China	244	6.15	0.00	93.85	0.23
Yellow river basin	53	41.51	0.00	58.49	0.68
South China	110	8.18	2.73	89.09	0.41
Korea	17	0.00	0.00	100.00	0.00
Japan	22	0.00	0.00	100.00	0.00
Russia	17	0.00	0.00	100.00	0.00

### Northern blot and qRT-PCR analysis of miRNAs

**Fig. 4:**
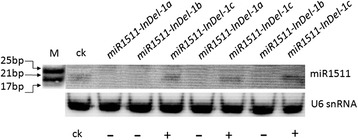
Small RNA northern blotting analysis of miR1511. Expression of miR1511 in various accessions of *G. soja*, M: marker; CK: control; deletion of miR1511 (*miR1511-InDel-1a*); partial deletion of miR1511 (*miR1511-InDel-1a*); present of miR1511 (*miR1511-InDel-1c*)

We experimentally verified the presence and absence of miR1511 by Northern blotting using specific probes and U6 snRNA as an internal reference. We randomly selected three accessions each which representing absence and presence of miR1511 in *G. soja* and Zhongpin 95–5383 *(G. max)* as control (C) (Fig. 4). In accessions without the deletion of miR1511 and in the control (*G. max*), we detected the signal at 21 bp, but no signals were detected in accessions with complete deletions of miR1511 indicating there is no expression in miR1511-deleted accessions as compared to *G. max* and accessions with no deletions.

**Fig. 5:**
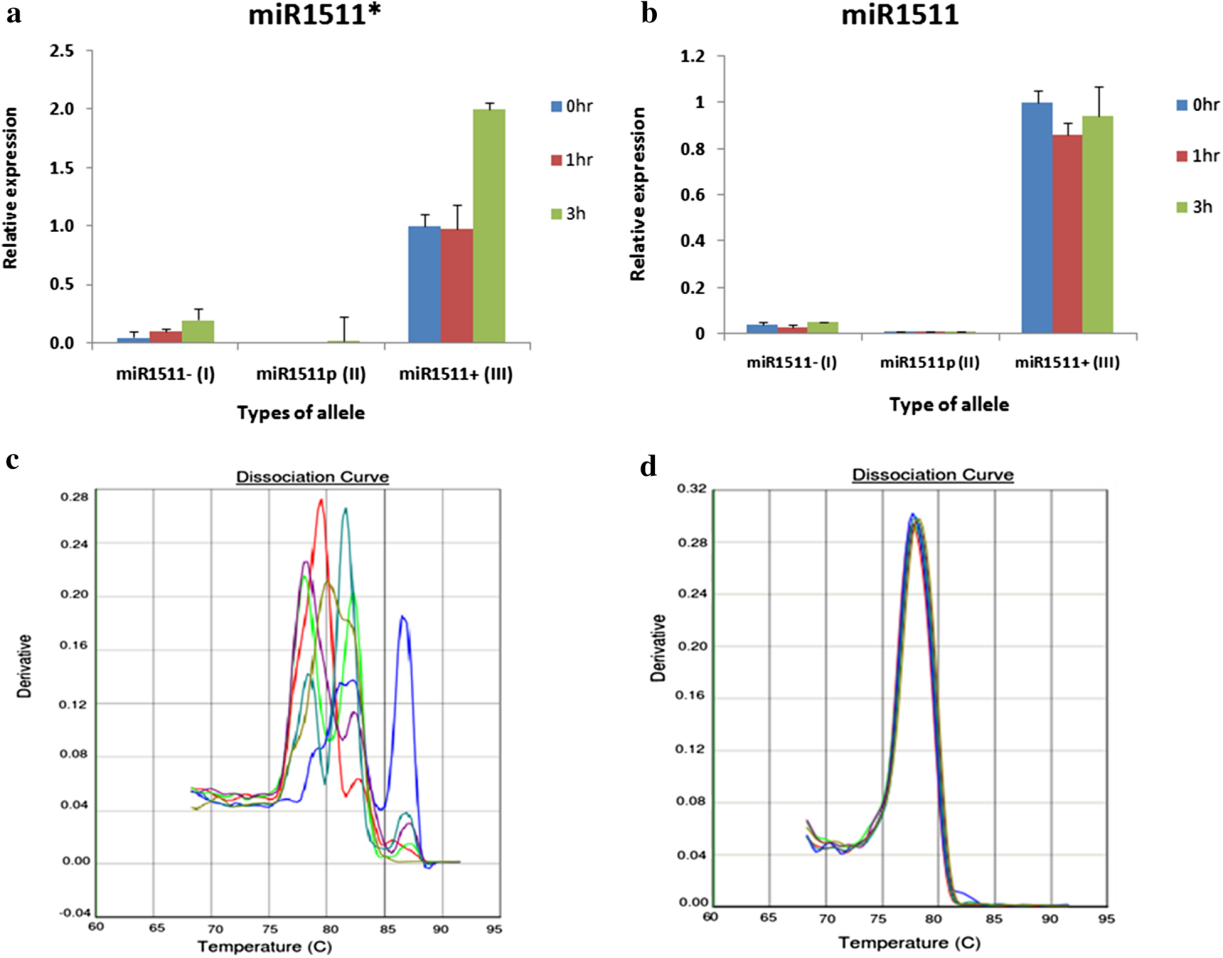
qRT-PCR analysis of miR1511 and miR1511* under drought stress. Expression of miR1511 and miR1511* in different accessions of annual wild soybean. (**a**) expression of miR1511 (**b**) expression of miR1511* (**c**) dissociation curve with multiple peak using Type I and Type II accessions in miR1511 (**d**) dissociation curve with only one peak using Type I, II and III accession in miR1520d as an internal control. Error bars represent standard error for three replicates. miR1511- (I): *miR1511-InDel-1a* (deletion), miR1511p (II): *miR1511-InDel-1b* (partial deletion*)*, miR1511+ (III): *miR1511-InDel-1c* (no deletion)

In addition, we further provided the present or absent of miNA1511 and miR1511* by qRT-PCR under drought stress in 0, 1, 3 h time points respectively. Both miR1511 and miR1511* expressed only in Type III (*miR1511-InDel-1c*) accession under both control and drought stress condition, and no transcription for type I (*miR1511-InDel-1ab*) and type II (*miR1511-InDel-1b*) accessions in all conditions (Fig. 5a, b). The melting curve analysis showed two separate peaks for Type I (*miR1511-InDel-1a*) and Type II (*miR1511-InDel-1b*) accessions at real-time PCR dissociation curve that was completely irreproducible for both miR1511 and miR1511* transcriptions (Fig. 5c). However, there was only one peak for miR1520d which was used as internal control in type I (*miR1511-InDel-1a*) and Type II (*miR1511-InDel-1b*) accessions (Fig. 5d). The most possible cause presenting two separate peaks at dissociation curve is non-specific amplification which clearly indicated that no function in type I (*miR1511-InDel-1a*) and type II (*miR1511-InDel-*1b) accessions which lack both of miR1511 and miR1511*. This data is also consistent with northern blot confirmation which only showed the signal at 21 bp in type III (*miR1511-InDel-1c*) accessions (Fig. 4).

## Discussion

### MicroRNA functional markers—miR1511 as an exemplar

Mapping markers have evolved from morphological markers to isoenzyme marker to random DNA markers and now to gene targeted functional markers developed from polymorphic sites within genes. Although the use of some recently developed marker techniques in plant sciences is not yet as extensive as that of well established methods such as SSR markers, the number of studies utilizing these advanced methods is rapidly increasing [5]. In this report, we demonstrate four interesting features of miR1511-InDel marker. Firstly, miR1511-InDel is part of a new class of small RNAs (miRNAs) that are single copy number and from which we were able to make inferences about genetic diversity within the subgenus Glycine. Secondly, marker developed from miRNA can be developed easily with efficient detection though detection of miRNA is difficult. It is evidence that this miR1511-InDel marker could be distinguished on the presence or absence of miR1511 due to allelic variation within the pre-miR1511 in soybean germplasm. Thirdly, miR1511-InDel is a functional marker as miRNAs play in functional roles in the regulation of gene expression at the post-transcriptional level in eukaryotes and viruses [26, 36]. In addition, it has been confirmed that there are anti co-expression between miR1511 and *GmRPL4a* in different tissue [35]. In addition, there are anti co-expression between miR1511/miR1511* and *GmRPL4a* under drought treatment (Additional file 4: Figure S4). Recently, it has been reported that loss-of-function mutants of *RPL4A*, rpl4a, which were initially identified as a mutant with altered trafficking of vacuolar targeted proteins, display auxin-related developmental defect phenotypes such as narrow pointed first leaves and retarded growth [37]. Fourthly, miR1511-InDel is soybean-specific as confirmed by the existence of complementary strand of miR1511 (miR1511*) and verified by its target cleavage site (*GmRPL4a*) [35] yet found in any other crop. *GmRPL4a* belongs to 60S ribosomal protein L4 family and is homologous to *RPL4a* in *Arabidopsis* which is involved in plastid transcriptional regulation [38]. Furthermore, there was no sequence match for pre-miR1511 in *P. vulgaris* (Additional file 3: Figure S3) and the flanking region of target gene cleavage site in *P. vulgaris* was diverged from that of soybean. Moreover, there was no sequence match to mature miR1511 in *P.valgaris* and there were three SNPs at miR1511* (Additional file 5: Figure S5).

### Mode of miRNA evolution or producing

The first miRNAs, Lin-4 and Let-7, were discovered as the components of developmental pathways in *Caenorhabditis elegans* [39–41]. Since then many studies have found and elucidated the roles of miRNAs in diverse species, including plants. The evolutionary history of miRNA genes has been well characterized in animals, for example the formation of the miR-17 cluster [42] and the imprinted miR-134 gene cluster at human locus 14q32 [43–45]. With the increasing number of identified miRNAs by bioinformatics prediction and deep sequencing, species specific or non conserved miRNAs have been found in rice [46], *Medicago truncatula* [47] and soybean [31]. It has been suggested that as the majority of these are restricted to closely related species and many could be species specific. Therefore, it is reasonable to hypothesize that plants harbor relatively large numbers of recently born miRNA loci [48]. In plants, some miRNAs and their target genes have been conserved since the last common ancestor of bryophytes and seed plants more than 400 million years ago [49]. Evolution of miRNA genes in plant are thought to originate via three modes: i) produced by inverted duplication of target gene sequences [50]; ii) generated from inverted repeat transposable elements [51]; and iii) spawned from random foldback sequences (“spontaneous evolution”) [52]. In this study, the miR1511 foldback lack extended similarity or complementary to the target gene, *GmRPL4a*, [35] or any other of the predicted target gene sequence. Therefore, it is inconsistent with the hypothesis of inverted duplication in which the foldback arms of recently evolved miRNA genes containing relatively long sequences similar to target gene sequences [50]. Transposable element-derived miRNAs have the potential to regulate multiple genes via homologous target sites dispersed throughout the genome [51]. The target gene of miR1511 has another copy, *GmRPL4d*, that was not cleaved by miR1511, as determined by 5' RACE [35], and thus may not be derived from a transposon. It is possible that miR1511 could be derived from random foldback sequences. Thus, by chance, miR1511 could be a newly spawned and species-specific miRNA as even some of the closely related wild species, *miR1511-InDel-1a*, lack the mature miR1511 sequence (Fig. 1).

### Diversity or natural selection of miR1511

It has been established that cultivated soybean (*G. max*) was domesticated from its annual wild relative soybean **(***G. soja*) in East Asia more than 3,000 years ago [53]. SSR, SNP and resequencing indicated that *G. soja* has greater genetic diversity and higher allelic diversity than *G. max* [16, 54]. Similarly, in this study we found that marker miR1511-InDel can differentiate between the subgenus of Glycine and Soja in which allelic diversity was found only in *G. soja* (Table. 1). On the basis of the geographical distribution of accessions, the Yellow River basin (Huanghuai) is the most likely centre of diversity where allelic variation, especially as the complete deletion of mature miR1511 in *miR1511-InDel-1a*, was observed mostly in that region (Fig. 3). Moreover, our data is consistent with pan genome analysis of seven wild soybean accessions (*G. soja* A to G) accessions [18] in which only *G. soja* D from Yellow River basin (Huanghuai) had a complete deletion of mature miR1511. According to phylogenetic tree (Fig. 2), we hypothesize that miR1511 is missing in the most ancestral form of *G. soja* and was gained in later *G. sojas* and then passed through the domestication bottleneck to the domesticated *G. max*. It has been suggested that domestication of cultivated soybean was directional selection as soybean is a short- day plant that is adapted to a narrow geographic range, accessions adapted to certain geography would have difficulties in adapting to various eco-regions in breeding especially for yield-related traits [55]. In addition, there were two whole genome duplication events in soybean [2], and with subsequent gene loss. This study attempted to infer the genetic variation and evolutionary relationship within the genus Glycine using a functional miRNA marker.

### Origin of soybean genetic diversity

Soybean originated from China where the cultivation of soybean has a long history of more than 5000 years, notably the agricultural ancestor Houji planted five crops including soybean [56]. Many scholars have pointed out different geographic origins of soybean in China including the Northeast region, the Yellow River valley region (Huanghuai) and the South region [57–59]. In this study, the phylogenetic tree analysis of allelic variants from miR1511 allelic pointed out that partial or non missing forms of miR1511 in *miR1511-InDel-1b* and *miR1511-InDel-1c* are likely to have originated from the missing form of *miR1511-InDel-1a* which was mainly distributed in the Yellow River basin (Huanghuai) (Fig. 3). Therefore, we suggest that the Yellow River basin (Huanghuai) is the primary centre of species diversity as supported by miR1511. Similarly, the highest diversity has been observed for ten quality traits and five quantitative traits and SSR analyze in seven clusters of 23,587 soybean landraces [59] in this region. A SSR and SNP analyses in 303 accessions of domesticated soybean and its wild progenitor indicated that *G.max* was originated from regions along the Yellow River of China [16]. Thus, the distribution of allelic diversity of miR1511-InDel provides more molecular evidence for the domestication of soybean in the yellow river basins (Huanghuai).

## Conclusions

In conclusion, the functional miR1511 marker (miR1511-InDel) was localized within a functional region and found to be useful to analyze genetic variation within Glycine. This was done using simple equipment and standard conditions with low cost. The marker can also be used as a tool for further analysis of phylogenetic relationships using the flanking region of miR1511 in this crop and those of model legumes. Our study suggested that the InDel marker of miR1511 can help to provide a reference for studying genetic diversity, genotyping of germplasm, evolution and the center of species diversities in domestication of soybean and that this marker system could be useful for many applications in breeding and ecology/evolution.

## Methods

### Plant materials

We collected a total of 1,669 accessions representing cultivated soybean [*G. max* (L.) Merr.] (1,206) (Additional file 6: Table S1) and wild progenitor species [*G. soja* Sieb. et Zucc] (463) (Additional file 7: Table S2). The cultivated soybean including the minicore collection [60] were collected from Northeast region, North region, Huanghuai region (Yellow River basin) and South region and 10 out-group accessions collected outside of China. The wild progenitor species originated from three geographical regions of China, Japan, Korea and Russia. Four perennial wild soybean *Glycine microplylla, Glycine tabacine, Glycine latifolia and Glycine tomentella* were used for post-PCR sequence alignment. All the accessions within China were selected to represent the geographical range from 23.7 to 51.4° N and 106.4 to 131.5° E.

### Bioinformatics data and data analysis

Soybean genome sequences were obtained from the Phytozome database (http://www.phytozome.net/). The Primer Premier Version 5.00 software package (Premier Biosoft International) was used to design primers and Multalin (http://multalin.toulouse.inra.fr/multalin/multalin.html) for sequence alignments. An unrooted phylogenetic tree was constructed using MEGA 4.0 with the maximum likelihood (ML) method and bootstrap tests carried out with 1000 iterations [61]. The predicted sequence of pre-miR1511from other crops were confirmed for present of mature sequences and their secondary structure predicted using the RNAfold program (http://mfold.rna.albany.edu/?q= mfold). The Shannon diversity index was calculated as H = −sum (Pi ln[Pi]) where H represents the Shannon diversity index and Pi is the number of individuals in a particular species type divided by the total number of individuals of all types of species in the community.

### Genomic DNA extraction and sequencing

Genomic DNA was extracted using Genomic DNA Purification Kits (Thermo Scientific (Cat # K0512) according to manufacturer’s protocol. All the extracted DNA samples were normalized to 20 ng/μl for PCR amplification. MIR1511 gene fragment amplification primer sequences were designed as follows: MIR1511F, 5'-TCTTCATG GACTTATTTGCCACTTC- 3'; MIR1511R, 5'- CCTTCAAGACCAAATGCTAAA T C G- 3'. The PCR reactions performed with Ex Taq kit (Takara,Dalian,China) with the following cycling parameters: initial denaturing at 94 °C for 4 min, 35 cycles of denaturing at 94 °C for 30 s, annealing at 56 °C for 30 s, extension at 72 °C for 30 s and final extension at 72 °C for 8 min. The PCR product was detected by electrophoresis on 1.5 % agarose gels and the expected fragments were recovered by using a DNA gel extraction kit (Axygen Biotechnology, Hangzhou, China). The purified fragments were ligated into the pMD18-T vector (TaKaRa, Dalian, China) at 16 °C for 1 h followed by transformation into *Escherichia coli* Top10 competent cells (Tiangen Biotech, Beijing, China) and spread onto LB agar plates containing 100 μg/mL ampicillin. The plates were incubated at 37 °C for 12–16 h. Randomly selected colonies were cultured in liquid LB medium containing 100 μg/mL ampicillin at 37 °C on an oscillator for 6 h. Positive recombinant clones were screened by colony PCR. PCR products containing the expected inserts were sequenced using M13 forward and reverse primers (GENEWIZ Beijing, China).

### RNA isolation and Northern blot analysis

Total RNA was extracted using an RNAiso plus kit (Takara, Dalian, China) according to manufacturer’s protocol and small RNA enrichment was performed as previously described [62]. 40 μg of enriched small RNA was separated in a 15 % polyacrylamide gel with 8 M urea, in MOPS buffer (pH 7.0), along with the microRNA marker (New England Biolabs, Ipswich, UK). The samples were electroblotted to positively charged nylon membrane (Amersham Life Science, Buckinghamshire, UK) using a semi dry transfer cell (BioRad Laboratories, Richmond, CA, USA). The nylon membrane was again placed onto the Stratagene UV Cross linker (UPV, San Gabriel, CA, USA) at 1200 kJ (front, back and front for 1 min each), baked at 80 °C for 30–60 min and then prehybridized at 42 °C in ULTRAhyb- Oligo buffer (Ambicon the membrane at, Austin, TX, USA) for 2–4 h in a standard rotating hybridization oven. To detect miRNA1511, short oligonucleotides probe (Nor_1511), 5'-CCATGGTATCAGAGCCT GGTT- 3'; the U6 snRNA probe (Nor_U6), 5'- GACCATTTCTCGATTTGTGCG TGTC- 3' were end-labeled with γP^32^-ATP (PerkinElmer, Waltham, MA, USA) overnight at 37 °C, using T4 polynucleotide kinase (USB Corp, Cleveland, OH, USA). After hybridization, the membrane was washed at 42 °C using a 2xSSC + 0.5 % SDS solution until radioactivity was reduced to less than 300 cpm. The hybridized membrane was exposed to a storage phosphorimager screen (GE Healthcare, Milwaukee, WI, USA) and scanned using FX Pro Plus (BioRad Laboratories, Hercules, CA, USA).

### Quantitative real-time PCR

The following accessions were used for *miR1511* and miR1511*gene expression analysis. Type I (*miR1511-InDel-1a*) ZYD03386, which loss mature sequence of miR1511, type II (*miR1511-InDel-*1b) ZYD04366 which loss partial sequence of miR1511 and miR1511* and type III (*miR1511-InDel-1c*) ZYD03960 which have miR1511 and miR1511* were used. Total RNA were extracted from the leaves of V2 stage of seedlings treated by drought (paper dry) at 0, 1, 3 h respectively using RNAiso Plus (Takara, Dalian, China) reagent followed by the manufacturer’s instructions.

First-strand cDNA synthesis of miRNA was then performed using a miRcute miRNA first-strand cDNA synthesis kit (Tiangen, Beijing, China) according to the manufacturer’s instructions. Relative quantification of miRNA expression was carried out using PRISN 7300 real- time PCR system (Applied Biosystems, Foster City,CA, USA) with SYBR Green miRcute miRNA qPCR Detection Kit (Tiangen, Beijing, China) which contained antisense adaptor primers and applying the corresponding miRNA sequences as sense primers. Soybean miR1520d was used as an internal standard. The data were analyzed using the 2^–ΔΔCt^ method. The PCR primers for qPCR of miR1511, miR1511* and miR1520d were as follows: miR1511 primer (5′- GGAACCAGGCTCTGATACCATGG −3′), miR1511* (5′- CCGTGGTATCAGG TCCTGC TTCA-3′) and miR1520d primer (5′ ATCAGAACATGACACGTGACAA-3′). The experiment was repeated three times.

### Availability of Supporting Data section

All the supporting data are included as additional files.

## Additional files

Additional file 1: Figure S1. Predict sequence of stem loop miR1511. Predict sequence of stem loop miR1511 in various organisms, underline represents mature sequence.

Additional file 2: Figure S2. Stem loop miR1511 structure by RNA folding in various organism. Pre miRNA stem loop structure by RNA folding (http://mfold.rna.albany.edu/?q=mfold) (a) *L. japonica* (b) *G. max* (c) *P. tricocarpa* (d) *M. truncatula* (e) *V. vinifera.*

Additional file 3: Figure S3. Alignment of precursor of miR1511 with pan-genome accessions. Alignment of precursor sequence of miR1511 in cultivated soybean *G. max*, seven *G. soja* accessions in pan- genome and *P. vulgaris.*

Additional file 4: Figure S4. Anti expression of miR1511/miR1511* and its target gene *GmRPL4a* under stresses condition. QRT-PCR was performed using Type III, *miR1511-InDel-*1c accessions (a) expression of miR1511* and miR1511 under drought stress (b) expression of *GmRPL4a* under drought stress [target cleavage site of both miR1511* (GmRPL4*) and miR1511 (*GmRPL4*)] (c) expression of miR1511* and miR1511 under salt stress (d) expression of *GmRPL4a* under salt stress [target cleavage site of both miR1511* (GmRPL4*) and miR1511 (*GmRPL4*)]. Error bars represent standard error for three replicates.

Additional file 5: Figure S5. Alignment of miR1511 target gene with pan-genome accessions. Alignment of miR1511 target gene among *G.max*, seven *G.soja* from pan genome and *P. vulgaris* (a) tail region of the target gene (b) miR1511* complementary in target gene region (c) miR1511 complementary in target gene region.

Additional file 6: Table S1. List of cultivated soybean. List of the cultivated soybean (*G. max*) accessions used in this study.

Additional file 7: Table S2. List of wild soybean. List of the annual wild soybean (*G. soja*) accessions used in this study. I: *miR1511-InDel-1a*; II: *miR1511-InDel-1b*; III: *miR1511-InDel-1c.*
