# Supplementary material for: Functional marker development of miR1511-InDel and allelic diversity within the genus Glycine
Source: BMC Genomics. 2015 Jun 18;16(1):467. doi: 10.1186/s12864-015-1665-3 (PMC4470002; doi:10.1186/s12864-015-1665-3)
Supplement: Supplementary file 1 — Predict sequence of stem loop miR1511. Predict sequence of stem loop miR1511 in various organisms, underline represents mature sequence. [file 12864_2015_1665_MOESM1_ESM.doc]

**Additional File 1: Figure 1**

> gi|387762943|ref|NR_048619.1| *Glycine max*

TCAGCCGTGGTATCAGGTCCTGCTTCATCAAGTGGTCTTGTGTTCAAATCCAGCCTCAAGCACATGGTTAACCAGGCTCTGATACCATGGTGAATATAA

> gi|41688314|dbj|AP006629.1|:11240-11259| *Lotus japonicus*

TCTATTCATGGTATCATAACCTGCTTCACTTAGTGGTCTTGTGTTCAAATACTGCTCAAGCACATGGTGAACCAGGCTCTGATACCATGAAGCGATTGG

> gi|158303386|gb|AC212803.1|:37466-37485| *Populus trichocarpa*

GACATTGGTGAACATGATTTCTACAGGGGCAGCGAGTTCCATAAAATTAGTAAGCCAAGGGTTCGACCAACCAGGCTCTGATACCATGTTAAAGTAAT

> gi|167963549|gb|AC146559.29|:119836-119855| *Medicago truncatula*

CTTATTTAACATGGTATCAGAGCCGGGTTAATGATTGCGATGTGGGCGCTTAGGTTCTCAGGTTGTCGAGTGTATAAATATGCTTGAGTATCCCTCCTC

> gi|123715102|emb|AM481601.1|:126128-126147| *Vitis vinifera*

CTTCTTAGATTCAACATGGTATCAGAGCCTTAGGCTCGTAAATATGGGAATCAAGAAGAAGAATTTTCAGCCTTCATTGTCGAGTTTGAAGATCAGAGTC
