# Supplementary figures and images for: Functional marker development of miR1511-InDel and allelic diversity within the genus Glycine
Source: BMC Genomics. 2015 Jun 18;16(1):467. doi: 10.1186/s12864-015-1665-3 (PMC4470002; doi:10.1186/s12864-015-1665-3)

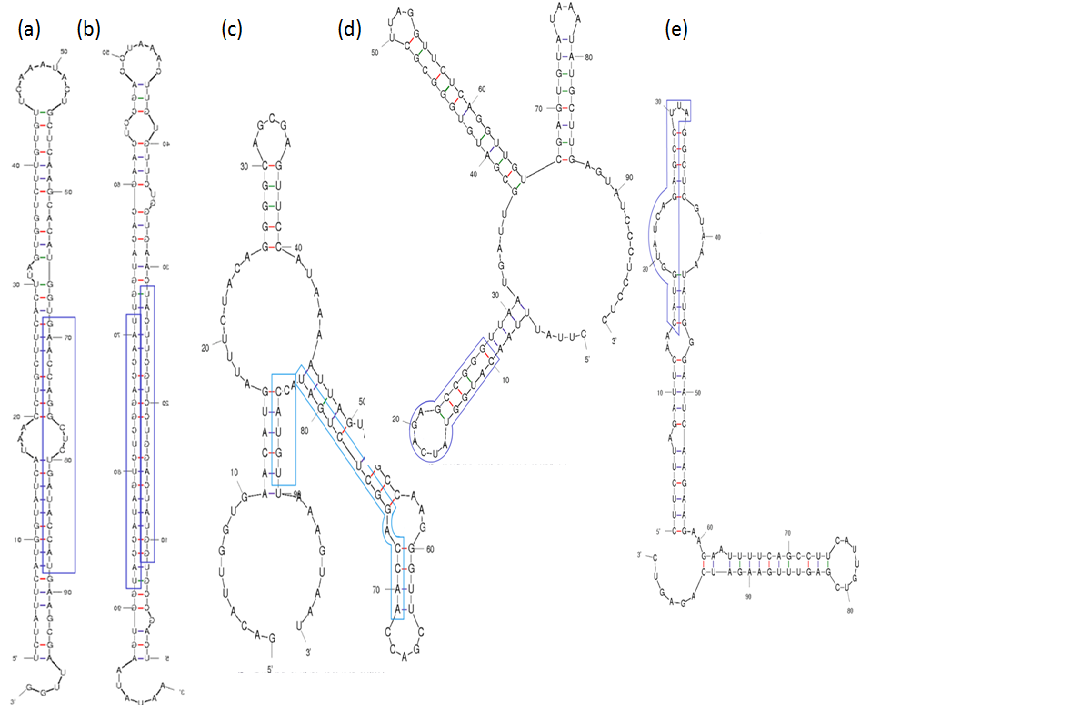

Supplement: Supplementary file 2 — Stem loop miR1511 structure by RNA folding in various organism. Pre miRNA stem loop structure by RNA folding (http://mfold.rna.albany.edu/?q=mfold) (a) L. japonica (b) G. max (c) P. tricocarpa (d) M. truncatula (e) V. vinifera. [file 12864_2015_1665_MOESM2_ESM.png]

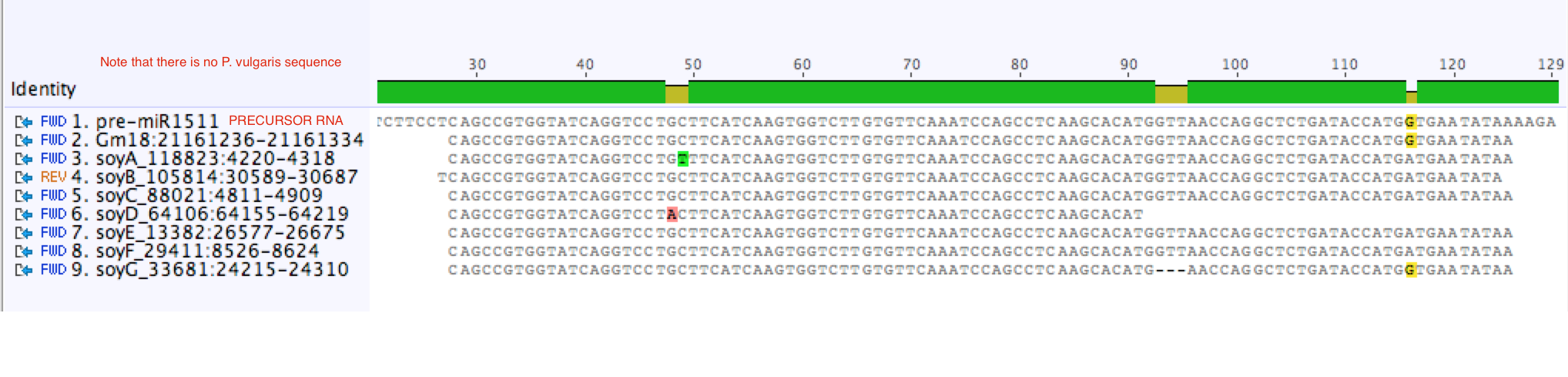

Supplement: Supplementary file 3 — Alignment of precursor of miR1511 with pan-genome accessions. Alignment of precursor sequence of miR1511 in cultivated soybean G. max, seven G. soja accessions in pan- genome and P. vulgaris. [file 12864_2015_1665_MOESM3_ESM.png]

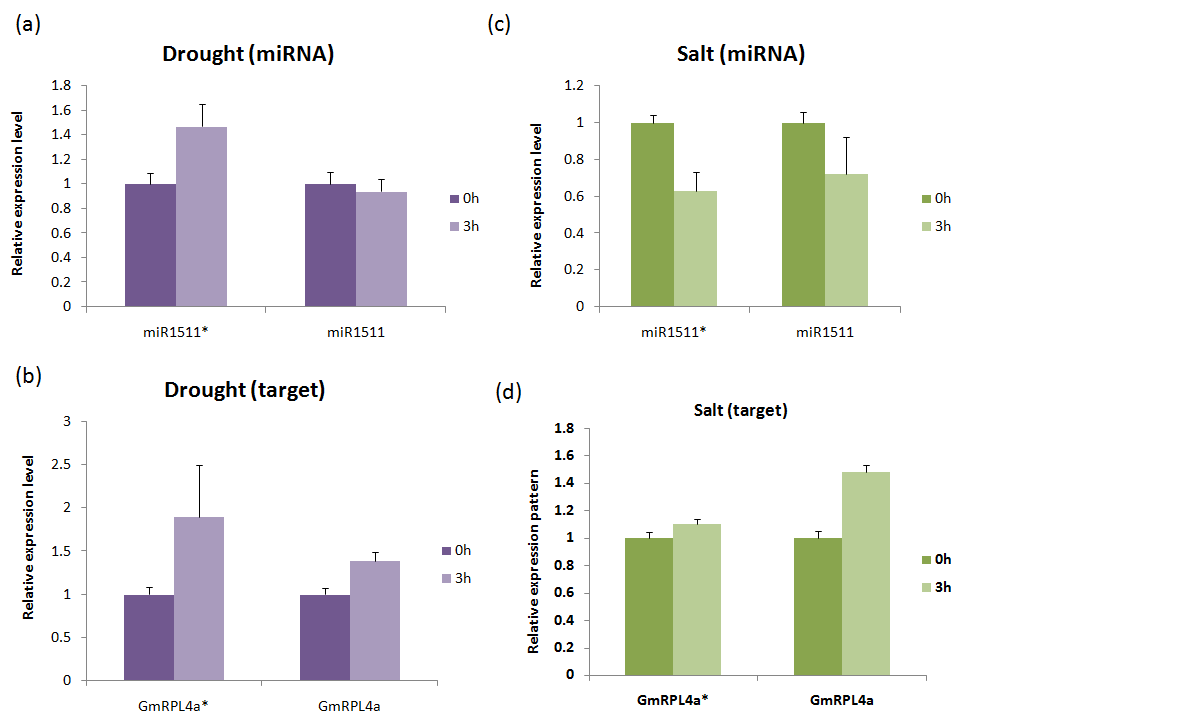

Supplement: Supplementary file 4 — Anti expression of miR1511/miR1511* and its target gene GmRPL4a under stresses condition. QRT-PCR was performed using Type III, miR1511-InDel-1c accessions (a) expression of miR1511* and miR1511 under drought stress (b) expression of GmRPL4a under drought stress [target cleavage site of both miR1511* (GmRPL4*) and miR1511 (GmRPL4)] (c) expression of miR1511* and miR1511 under salt stress (d) expression of GmRPL4a under salt stress [target cleavage site of both miR1511* (GmRPL4*) and miR1511 (GmRPL4)]. Error bars represent standard error for three replicates. [file 12864_2015_1665_MOESM4_ESM.png]

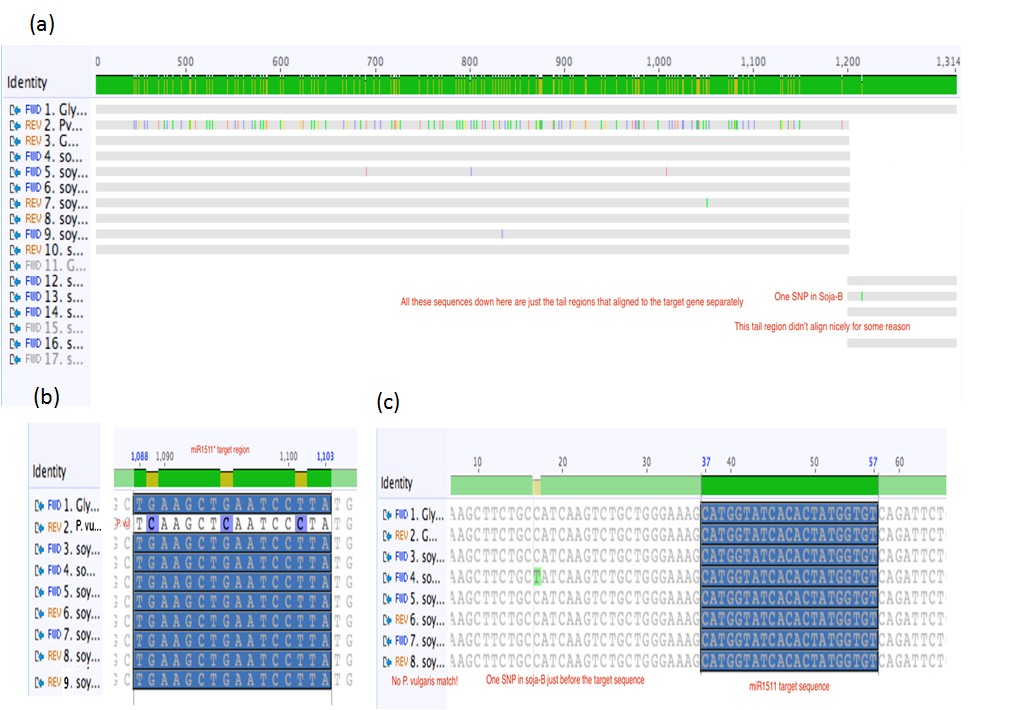

Supplement: Supplementary file 5 — Alignment of miR1511 target gene with pan-genome accessions. Alignment of miR1511 target gene among G.max, seven G.soja from pan genome and P. vulgaris (a) tail region of the target gene (b) miR1511* complementary in target gene region (c) miR1511 complementary in target gene region. [file 12864_2015_1665_MOESM5_ESM.png]
